# High-Performance Zwitterionic Organohydrogel Fiber in Bioelectronics for Monitoring Bioinformation

**DOI:** 10.3390/bios13010115

**Published:** 2023-01-09

**Authors:** Jun Xia, Jiabei Luo, Boya Chang, Chuanyue Sun, Kerui Li, Qinghong Zhang, Yaogang Li, Hongzhi Wang, Chengyi Hou

**Affiliations:** 1 State Key Laboratory for Modification of Chemical Fibers and Polymer Materials, College of Materials Science and Engineering, Donghua University, Shanghai 201620, China; 2 Engineering Research Center of Advanced Glasses Manufacturing Technology, Ministry of Education, College of Materials Science and Engineering, Donghua University, Shanghai 201620, China

**Keywords:** bioelectronics, zwitterion, organohydrogel fiber, bioinformation monitor

## Abstract

Bioinformation plays an imperative role in day-to-day life. Wearable bioelectronics are important for sensing bioinformation in real-time and conductive hydrogel fibers are a key component in next generation wearable bioelectronics. However, current conductive hydrogel fibers have remarkable disadvantages such as insufficient conductivity, stability, and bioinformation sensing ability. Here, we report the synthesis of a zwitterionic organohydrogel (ZOH) fiber by the combination of the mold method and solvent replacement strategy. The ZOH fiber shows transparency (92.1%), stretchability (905.8%), long-term stability, anti-freezing ability (−35–60 °C), and low light transmission loss (0.17 dB/cm). Then, we integrate the ZOH fiber into fabric for use as a bioinformation sensor, the results prove its capability as a bioinformation monitor, monitoring information such as motion and bioelectric signals. In addition, the potential of the ZOH fiber in optogenetic applications is also confirmed.

## 1. Introduction

Bioinformation, such as genetic, neurological, chemical, and sensory information, plays an essential role in reflecting, regulating, and controlling day-to-day activities [1,2,3]. With the increasing attention paid to bioinformation, the importance of bioelectronics that can detect and deliver bioinformation has become increasingly prominent. For bioelectronics, the realization of highly sensitive, real-time, and multi-functional detection has become the jewel in the crown of bioinformation detection [4,5,6].

Due to their tissue-like properties, conductive hydrogels are regarded as a critical material for bioelectronics [7,8,9,10,11]. Nowadays, conductive hydrogels are mainly divided into electron-conductive hydrogels and ion-conductive hydrogels [12]. However, both of these have shortcomings that cannot be ignored. For electron-conductive hydrogels, the main disadvantages are as follows: (1) the insufficient transparency due to the addition of commonly opaque electron-conductive fillers; (2) the limited elongation at break due to the inhomogeneous agglomeration caused by electron-conductive fillers; and (3) the poor fatigue resistance properties due to the fragile and unrecoverable bonding constructed by electron-conductive fillers [13,14,15,16]. For ion-conductive hydrogels, the main disadvantages are as follows: (1) the poor conductivity due to the restricted directional movement of freely charged ions in the hydrogel network and (2) the risk of ion leakage during the deformation of hydrogels due to high ion concentrations [17,18,19].

In addition to a performance which can overcome the disadvantages of electron-conductive and ion-conductive hydrogels, zwitterion-conductive hydrogels, which have an excellent biocompatibility, have come to the center of bioelectronics research [20,21,22]. For example, Hu and co-workers prepared a self-association cross-linked zwitterion-conductive hydrogel by simple copolymerization of carboxylbetaine methacrylate (CBMA) and hydroxyethyl methacrylate (HEMA), and applied it to the transmission of electrophysiological signals [23]. Wang and colleagues have developed zwitterionic hydrogels based on zwitterionic [2-(methacryloyloxy) ethyl] dimethyl-(3-sulfopropyl) ammonium hydroxide (SBMA) and polyvinyl alcohol, which could accurately and reliably detect subtle physiological signals, such as segment bending and wrist bending [24]. However, the long-term instability and insufficient temperature tolerance of the zwitterion-conductive hydrogels remain to be resolved.

In view of the above problems of zwitterion-conductive hydrogels, organohydrogels, which use organic compounds as a solvent, should be considered as an effective solution [25,26]. For example, Yang and co-workers prepared a physically cross-linked ion-conducting organohydrogel by introducing ethylene glycol (EG) as the organic solvent, which could remain unfrozen and flexible at low temperature (−40 °C) and exhibited long-term stability (12 days) [27]. Gao and colleagues used glycerol to prepare a conductive organohydrogel with significant anti-freezing (−20 °C) and water retention abilities (7 days) [28]. Ye and co-workers prepared an organohydrogel by using dimethyl sulfoxide (DMSO), which showed excellent anti-freezing abilities (−70 °C) and long-term stability (1 day) [29]. However, the organohydrogels reported in these works still have some obvious drawbacks, such as the limits of detectable bioinformation and the difficulty in integrating them with clothing to achieve wearability.

Herein, we creatively prepared a zwitterionic organohydrogel (ZOH) fiber based on the zwitterionic monomer [2-(methylpropenoyl) ethyl] dimethyl-(3-sulfopropyl) (SBMA) for strain sensors, flexible electrodes, and biofibers via the combination of the mold method and the solvent replacement method. The zwitterionic hydrogel (ZH) fiber was polymerized by UV light and then immersed in EG and LiCl solution for replacement to obtain the ZOH fiber. The ZOH fiber has ultra-high tensile properties (905.8%), high transparency (92.1%), a wide working range (−35–60 °C), and a high ionic conductivity (0.54 S/m). Through the solvent replacement method, the ZOH fiber obtains long-term stability and excellent durability. At the same time, the ZOH fiber can be used for detecting high-quality, stable electrocardiogram (ECG) and electromyogram (EMG) signals with low fluctuation. In addition, based on the high transparency of the ZOH fiber, we used it as a biofiber by the coating method, achieving an attenuation of 0.17 dB at the wavelength of 650 nm, demonstrating its potential for use in the field of optogenetics.

## 2. Materials and Methods

### 2.1. Materials

[2-(Methacryloyloxy) ethyl] dimethyl-(3-sulfonpropyl) (SBMA), sodium alginate (SA), and lithium chloride (LiCl) were purchased from Shanghai McLean Biochemical Technology Co., Ltd. Acrylamide (AAm), N, N’-methylenebisacrylamide (MBAA, 97%), and 2-hydroxy-4’-(2-hydroxythoxy)-2-methylpropiophenone (I2959) were purchased from Shanghai Aladdin Reagent Co., Ltd. (Shanghai, China). The inner diameter of the silicone hose was 0.8 mm, which was purchased from Kewenxin hardware store.

### 2.2. Preparation of the ZOH Fiber

Firstly, a certain amount of sodium alginate solution with a mass fraction of 1.5 wt% was prepared, and 3.88 g SBMA, 2.13 g AAm, 0.003 g MBAA, and 0.035 g I2959 were added in turn. The mixture was stirred until dissolved, stirred for 1 h until the solution was uniform and transparent, and stood for 6 h for defoaming. The obtained solution was then injected into a PVC tube with an inner diameter of 0.8 mm and irradiated with an ultraviolet lamp (photopolymerization at λ = 365 nm) for 10 min. Hydrogel fibers could be easily removed from the PVC tubes. The obtained ZH fibers were immersed in 1.5 M LiCl and 50 wt% EG mixed solution for a period of time, and finally the ZOH fibers were obtained.

### 2.3. Mechanical Tests

The tensile test was performed by a universal material testing machine (INSTRON, Boston, MA, USA) at room temperature. A tensile load sensor of 100 N was used to carry out the tensile test on the fibers with a diameter of 0.8 mm and length of 5 mm. The measurement was repeated three times for each fiber and the average value was calculated. The tensile rate was fixed at 20 mm/min. Tensile stress (σ) was obtained by dividing the tensile stress (F) by the cross-sectional area (S). The calculation formula of elongation at break is ε = (L − L_0_)/L_0_, where L_0_ is the length of the ZOH fiber before stretching and L is the length of the ZOH fiber after stretching. The elastic modulus (E) was obtained by calculating the slope of the stress–strain curve.

### 2.4. Electrical Tests

The ionic conductivity of the ZOH fiber was measured by AC impedance spectroscopy (PEIS) in the frequency range of 0.1–1 × 10^3^ Hz. The ZOH fiber was cut into a single sample with a diameter of 0.8 mm and a length of 1 mm. This sample was fixed by copper tape at both ends of a glass slide, and then coated with silver paste. After the silver paste was cooled, the channel was formed. The resistance value was the intercept of Nernst diagram line with the horizontal axis, and the ionic conductivity was calculated by the formula σ = L/ (R ∗ S) (S: cross-sectional area of the fiber).

The two ends of the sensor were fixed on a stretching machine and connected with a digital multimeter (Keithley2400) by a copper wire. The initial distance between the two ends was 2 cm, and different stretching ratios were set to test the strain response of the sensor. In addition, by moving the sensor with a finger, the resistance change rate of the sensor changes with the different moving angles:

△R/R_0_ = (R − R_0_)/R × 100%

where R_0_ is the resistance without strain and R is the real-time resistance.

### 2.5. Long Term Stability Test

The stability of hydrogel fibers of different samples at room temperature was reflected by the weight of gel fibers obtained by the weighing method. The calculation formula is as follows:

Weight retention = W_t_/W_0_ × 100%

where W_t_ is the weight of fibers in real-time and W_0_ is the initial weight of the corresponding fiber.

### 2.6. Visible Light Transmittance Test

The sample was attached to the glass substrate, and using the transmission mode of an ultraviolet visible spectrophotometer, the transmittance of the sample at different wavelengths was tested in the wavelength range of 400–800 nm. The sample was a rectangular gel with a thickness of 2 mm.

### 2.7. Differential Scanning Calorimetry (DSC) Tests

Through DSC 8500, the sample was cooled in a crucible to −35 °C at a heating rate of 10 °C/min under a nitrogen gas flow, then the temperature was gradually increased to 40 °C, and its DSC curve was recorded.

### 2.8. Bioelectrical Signal Test

ECG signals were monitored from different electrodes through a multi-channel digital EEG instrument from Boruikang Technology Co., Ltd. (Shanghai, China) When measuring the ECG signal, the reference electrode was adhered to the lower leg and the working electrode was adhered to the finger of the right hand. The electrode based on the ZOH fiber was connected to the inside of the forearm for EMG signals. When processing ECG signals, the filtering frequency used was 10–30 Hz. When processing EMG signals, the filtering frequency used was 20–40 Hz.

### 2.9. Optical Measurements

Lasers (100 mw) with wavelengths of 405 nm, 515 nm, and 650 nm were coupled to the coated and non-coated ZOH fibers. Additionally, an optical power meter was used (PL-MW2000) to measure the power of transmitted light passing through the fiber (8 cm) and to determine the optical power attenuation through the coated and non-coated ZOH fibers.

## 3. Results and Discussion

**Figure 1:**
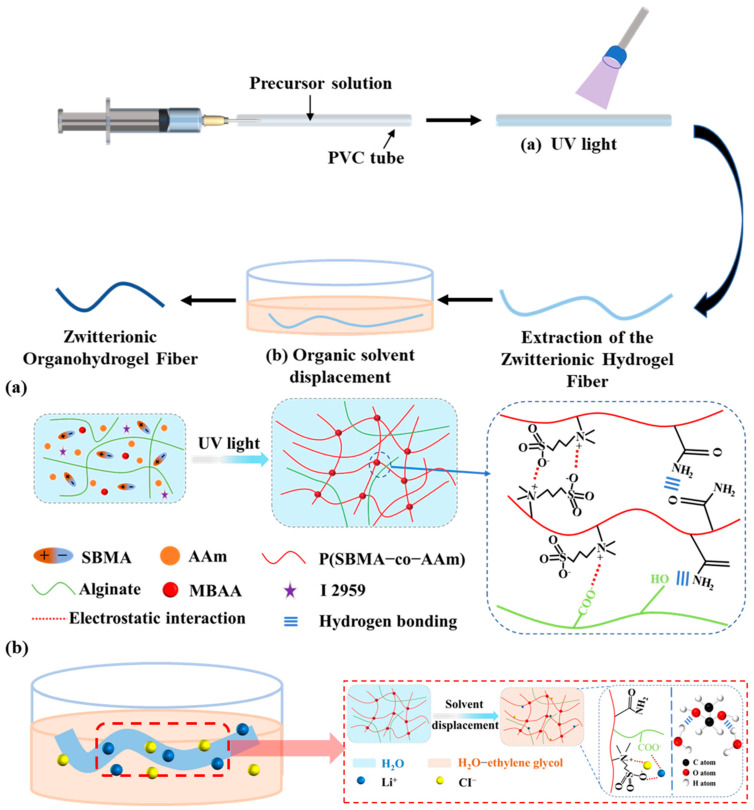
Fabrication strategy of the ZOH fiber. (**a**) Chemical structure of the ZH fiber. (**b**) Schematic of the preparation of the ZOH fiber from the ZH fiber by solvent displacement.

First, as shown in Table 1, we have marked the full names and abbreviations of materials and tests in the text. As shown in Figure 1, the ZOH fiber was obtained by the combination of the mold method and the solvent replacement method. MBAA and I2959 were used as the chemical crosslinking agent and initiator, respectively. AAm and SBMA were added to the dissolved sodium alginate solution, and this solution was injected into a PVC pipe for simple copolymerization. Then, the ZH fiber removed from the pipe was immersed in a mixed solution of LiCl and EG. After a period of time, the ZOH fiber was obtained.

In addition, Figure 1 shows a schematic illustration and microstructure of the ZH fiber (Figure 1a) and the ZOH fiber (Figure 1b). As shown in the blue dashed box, the P(SBMA-co-AAm) chain-to-chain sulfonic acid anions and ammonium cations have electrostatic interactions and additional intermolecular hydrogen bonding. Due to the rich carboxyl and hydroxyl groups in alginate, hydrogen bonds and electrostatic interactions can be induced in the P(SBMA-co-AAm) system to achieve double crosslinking, forming a semi-interpenetrating network in the P(SBMA-co-AAm) covalent crosslinking network. We have analyzed the structure of the ZH fibers by infrared spectroscopy. As shown in Figure S1, in the FTIR spectra of alginate and acrylamide, the peaks at 3339 cm^−1^ belong to the stretching vibration of –OH. Due to the addition of SBMA, an absorption peak at 1725 cm^−1^ appears, which is mainly due to the symmetric and asymmetric stretching vibration of the –SO_3_^−^ group. In the ZOH fiber with the addition of ethylene glycol (EG), a new absorption peak appears at 1087 cm^−1^. This absorption peak is due to the combination of hydroxyl groups on the ethylene glycol and groups in the chain, indicating the efficiency of the crosslinking reaction.

The ZOH fiber can be prepared by a simple solvent replacement. In simple terms, the prepared ZH fiber was immersed in a mixed solution of LiCl and EG, and the water of the ZH fiber was replaced by EG until equilibrium was reached. As shown in the red dashed box, a large number of hydrogen bonds were formed between the added EG, water, and the P(SBMA-co-AAm) chain, which improved the water retention of the hydrogel fiber, meaning it could be used long-term. The added LiCl has electrostatic interactions with the carboxylic acid anion, sulfonic acid anion, and ammonium cation in the alginate-P(SBMA-co-AAm) chain. LiCl not only improves the conductivity of the ZOH fiber, but also can retain the water in the ZOH fiber as an inorganic anti-freeze [30,31].

**Figure 2:**
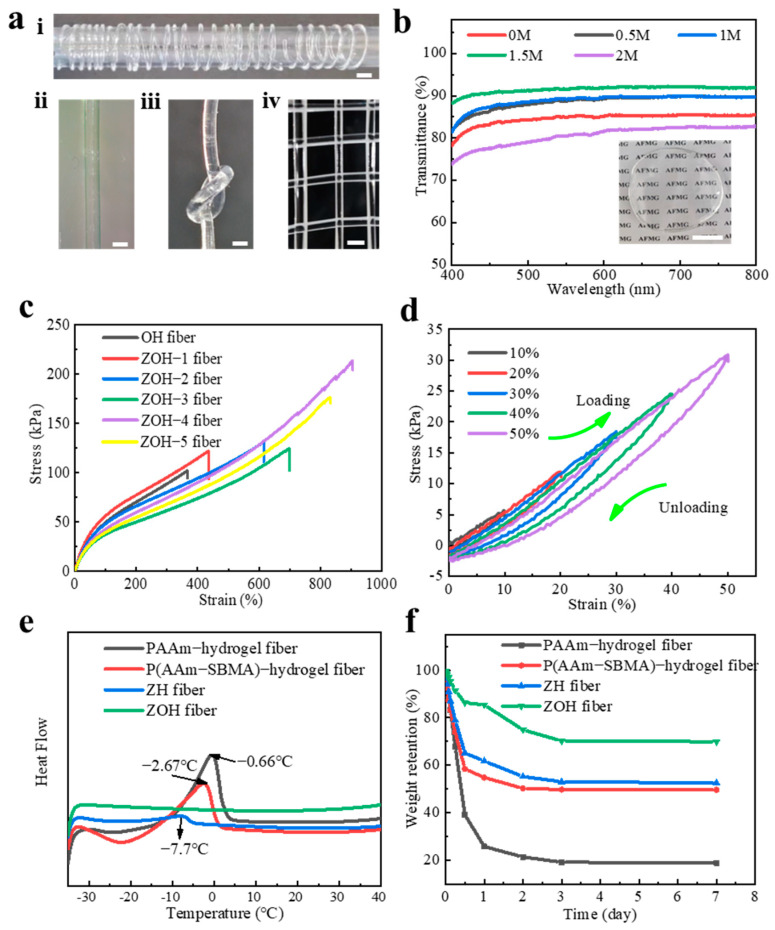
(**a**)(**i**) A representative photograph of a segment of the ZOH fiber. Scale bar: 1 cm. (**ii**) Image of the ZOH fiber under an super-depth-of-field microscope. Scale bar: 1 mm. (**iii**) Knotting ability of the ZOH fiber. Scale bar: 1 mm. (**iv**) A photo of the ZOH fiber forming a simple pattern. Scale bar: 2 mm. (**b**) Measured optical transparency of the ZOH fiber with different formulations in the spectral range of 400–800 nm. The illustration is a photo of a block of ZOH covering a pattern. Scale bar: 1 cm. (**c**) Tensile stress–strain curves for different SBMA contents. (**d**) Cyclic tensile stress–strain curves of the ZOH fiber with strains of 10%, 20%, 30%, 40%, and 50%. (**e**) The DSC curves of gels prepared with different formulations. (**f**) Long-term stability test of gels prepared with different formulations.

As shown in Figure 2a, a section of the ZOH fiber was wound on a white rod (i). Additionally, photos of the fiber were taken using an ultra-depth-of-field microscope. It can be seen that the prepared fiber has good morphology (ii). Additionally, the photos show the knotting ability of the ZOH fiber (iii). It can be seen that the ZOH fiber can be interlinked to form simple patterns (iv). In order to confirm the high transparency of the ZOH fiber, we took a rectangular zwitterionic organohydrogel (ZOH) film and used an ultraviolet–visible spectrophotometer to test the transmittance of the sample at different wavelengths in the wavelength range of 400–800 nm. The amount of LiCl added to the samples was controlled at 0–2 M and the transparency of a series of samples was tested. As can be seen from Figure 2b, the transparency of the samples increased as the LiCl content gradually increased from 0–1.5 M. The samples had the highest transparency of 92.1% when the LiCl content was 1.5 M. This may be due to the more complete reaction of LiCl with the ampholytic groups at this concentration. When the LiCl content was increased from 1.5–2 M, the transparency of the sample decreased, probably because the LiCl content was too high and a salting effect occurred, resulting in a decrease in transparency. The inset in Figure 2b shows a photograph of the ZOH fiber block covered with the pattern. The inset in Figure 2b shows a photograph of the ZOH fiber block covered with the pattern.

Organohydrogel fibers without SBMA and with molar ratios of SBMA to AAm of 1:16, 1:8, 1:4, 1:2, and 1:1 was prepared, named as OH fiber, ZOH−1 fiber, ZOH−2 fiber, ZOH−3 fiber, ZOH−4 fiber, and ZOH−5 fiber, respectively. By adjusting the content of SBMA, the mechanical properties of the ZOH fiber could be significantly affected. The elongation at break and mechanical strength of the ZOH−4 fiber were the best, with values of 905.8% and 213.4 Kpa, respectively (Figure 2c). Compared with other formulations, this formula had a lower Young ‘s modulus, indicating that the fiber has better skin compliance as it is closer to the modulus of human skin (Figures S2 and S4). If not specified, we used the ZOH−4 fiber with 1.5 M LiCl, as it was the best performing sample, for the following tests. We performed cyclic loading–unloading tensile tests on the ZOH fiber with different strains to prove the stability of the internal structure of the fiber. As shown in Figure 2d, there is a slight lag in the stress–strain curve under a small strain of 0~50% and a large strain of 50~400% (Figure S3). This may be because the network constructed by the P(SBMA-co-AAm) chains and alginate can dissipate energy well in the process of stretching. Due to the strong electrostatic force and hydrogen bonding force in alginate [32], the network structure could be maintained well, giving the ZOH fiber have good fatigue resistance properties.

Hydrogels often encounter extreme environmental environments, such as high-temperature dehydration and the low-temperature transformation of water into ice crystals, which can greatly affect the tensile properties and conductivity of hydrogels [33,34]. Therefore, most hydrogel-based sensors are prone to losing their sensing performance in extreme environments. With regard to this, the addition of EG and LiCl into the ZOH fiber reduced the freezing point of water via the strong hydrogen bonds and electrostatic interactions with the P(SBMA-co-AAm) chain [35,36], greatly enhancing the stability and the frost resistance of the hydrogels. Based on this, the ZOH fiber prepared by us can maintain good tensile properties and transparency at both low temperature (−35 °C) and high temperature (60 °C), while the ZH fiber prepared with water as the solvent is fragile at low temperature (−35 °C) and high temperature (60 °C), and the color of the fiber changes from transparent to opaque (Figure S5a,b).

In order to compare the frost resistance of different formulations of hydrogel fibers, we conducted a DSC test. As shown in Figure 2e, there was no peak between −35 and 40 °C for the ZOH fiber, while peaks do appear in this temperature range for other hydrogel fibers, indicating that the liquid–solid phase transition point of the ZOH fiber is higher than that of other hydrogel fibers. This is due to the addition of organic solvents and inorganic salts which broadens the phase transition range [37,38]. Then, we performed a thermogravimetric (TG) analysis test. As shown in Figure S6, the ZH fiber starts to decompose rapidly at 100 °C, while the ZOH fiber starts to decompose rapidly only when the temperature reaches 300 °C, which shows that the thermal stability of ZOH fiber is excellent. These data further demonstrate the possibility that the ZOH fiber can work in a lower temperature range.

Then, we tested the long-term water retention of the hydrogels with different formulations (Figure 2f). It can be seen that the weight of the pure acrylamide gel rapidly decreased, and there was a large water loss. Finally, the weight was only about 20% of its initial value. After a period of time, the final weight of the P(SBMA-co-AAm) hydrogels was about 50%, which may be due to the ion-dipole interactions between SBMA and AAm, which make the network structure of the gel more compact and traps the water in the gel. The alginate-P(SBMA-co-AAm) hydrogel formed a semi-interpenetrating network due to the addition of alginate, which increased the strong hydrogen bond interactions. However, the ZOH fiber formed hydrogen bond interactions and electrostatic interactions between chains due to the presence of EG and LiCl, which kept the weight of hydrogels above 70% and greatly ensured the long-term stability of the hydrogel.

**Figure 3:**
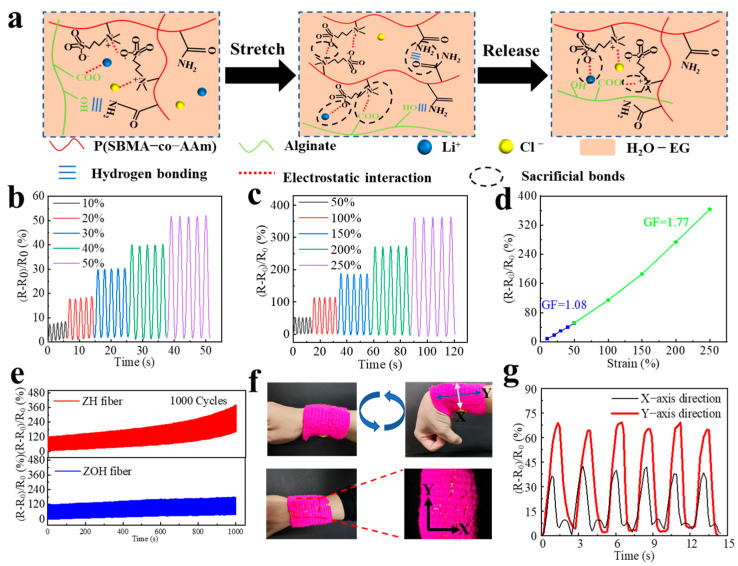
(**a**) Schematic of the evolution of a hybrid crosslinking network in the fiber during stretching. (**b**) Relative resistance changes in the strain sensor made from the ZOH fiber under small strains (10%, 20%, 30%, 40%, and 50%) cycles. (**c**) Relative resistance changes in the ZOH fiber-based sensor under large strains (50%, 100%, 150%, 200%, and 250%) cycles. (**d**) Relative resistance changes in ZOH fiber-based sensor as a function of strain. (**e**) Relative resistance changes in the ZOH fiber and the ZH fiber during cyclic stretching with a strain of 100% for 1000 cycles. (**f**) Photographs of the ZOH fiber woven into a fabric to create an anisotropic strain sensor and a wrist performing a bending motion. (**g**) The relative resistance changes in fibers in the X and Y directions with the bending and relaxing of the wrist.

As shown in Figure 3a, the dissipation mechanism of the ZOH fiber during stretching–unloading is described. The semi-interpenetrating network between the alginate macromolecular chain and the P(SBMA-co-AAm) chain, and the strong electrostatic forces between the lithium ions, the chloride ions and the chains greatly improve the elasticity of the fiber, which solves the problem of hysteresis of the fiber during stretching.

Then, we analyzed the sensing performance of the strain sensor made from the ZOH fiber. As shown in Figure 3b,c, the strain sensor can maintain a real-time, stable signal output under small cyclic tensions (10~50%) and large cyclic tensions (50~200%). It can be concluded that the relative resistance changes increase with the increase in strain, and the sensing coefficient (GF) was 1.77 in the large strain range (Figure 3d). In addition, in order to investigate the effect of different temperatures on the performance of this strain sensor we conducted the relevant tests, and it can be seen from the figure that the performance of the strain sensor remains stable under the influence of low and high temperatures (Figure S8). In addition to high sensitivity and accuracy, good fatigue resistance and potential for long-term use are essential requirements for the sensor [39]. Here, to demonstrate the long-term durability of the sensor based on the ZOH fiber, we used the sensor to perform 1000 tensile cycle tests at 100% strain under the same conditions. As shown in Figure 3e, after 1000 stretching cycles, the relative resistance change rate of the sensor changes greatly. This is due to the continuous loss of water in the fiber during the stretching–unloading process of the ZH fiber, which leads to an increase in resistance and the weakening of the hydrogen bonds between the chains, which worsens the elasticity of the fiber. The changes in relative resistance of the ZOH fiber are small after 1000 stretching cycles, and the output signal is stable because of the strong electrostatic interactions and hydrogen bonding inside the ZOH fiber. In addition, the EG within the fiber forms hydrogen bonds with water, which has a strong force, and the water is trapped inside the fiber. Thus, the elasticity and sensing performance of the ZOH fiber is maintained.

As a kind of elastic and highly stretchable fiber, the ZOH fiber can be used in optical fibers, bioelectric electrodes, strain sensors, and other applications. Therefore, we combined the ZOH fiber with a fabric wristband to form a fabric-type strain sensor. By placing the ZOH fiber orthogonally, different electrical signals are transmitted in both directions when the wrist bends (Figure 3f). It can be seen that the relative resistance change rate in the *Y*-axis direction is much larger than that in the *X*-axis direction (Figure 3g). This is because the strain of the wrist in the *Y*-axis is larger than the strain of the wrist in the *X*-axis.

**Figure 4:**
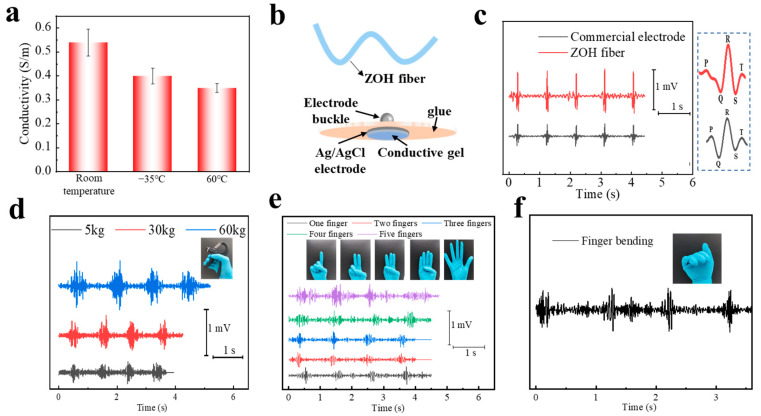
(**a**) Conductivity of the ZOH fiber at room temperature, −35 °C, and 60 °C. (**b**) Structure differences between ZOH fiber-based electrodes and commercial electrodes. (**c**) A comparison of ECG measurement results of commercial electrodes and ZOH fiber-based electrodes. The dashed frame shows the waveform of a single ECG signal. (**d**) EMG signals of varying gripping force detected by ZOH fiber-based electrodes. (**e**) EMG signals of different numbers of fingers extensions detected by ZOH fiber-based electrodes. (**f**) EMG signal of a knee bend detected by ZOH fiber-based electrodes.

In addition, we tested the conductivity of the ZOH fiber at different LiCl concentrations. It can be seen from Figure S7, that as the concentration of LiCl increased, the conductivity of the ZOH fiber gradually increased. The optimum conductivity was 0.62 S/m when the concentration of LiCl was 2 M. In addition, the ZOH fiber could maintain a high ionic conductivity of 0.4 S/m and 0.35 S/m at −35 °C and 60 °C, respectively (Figure 4a). At the same time, the ZOH fiber can be used as a flexible electrode for bioelectrical testing to obtain ECG signals. As shown in Figure 4b, we compared the structure of ZOH fiber-based electrodes and commercial electrodes. It can be seen that the ZOH fiber-based electrodes have a simple structure, and only a small section of fiber is required for accurate and real-time ECG signal measurements. The ECG signal of the ZOH fiber-based electrodes was compared with that of commercial electrodes.

It can be seen that the ECG signal peak measured by ZOH fiber-based electrodes is more obvious and the signal quality is better (Figure 4c), and the characteristic peak (PQRST) can be easily distinguished from the illustration in Figure 4c. The reason for the high quality of bioelectrical signals of ZOH fiber-based electrodes may be due to the high conductivity of the ZOH fiber, so the interface impedance combined with skin is small. We took a picture of the ECG signal being tested (Figure S9).

In addition, ZOH fiber-based electrodes can also be used for EMG signal tests. By placing a grip in a hand and changing the size of the grip, that is, the number of muscles to mobilize, we monitored the different grip transmission of the EMG signals. It can be seen from Figure 4d that with the increase in grip strength, the signal of the EMG gradually increased, there was no obvious fluctuation, and the signal remained stable. As shown in Figure 4e, by changing the number of finger extensions, due to the use of different strengths, the resulting EMG signal can also be well distinguished and the resulting EMG signal is stable. Figure 4f and Figure S10 show the EMG signal output when the finger and knee are bent. Figure S11 indicates the positions of ZOH fiber-based electrodes. In addition, as a flexible electrode in contact with biological epidermis, the ZOH fiber should have good biocompatibility. We set the cell viability of the control group to be 100%, and Figure S12 shows the cell viability results of the ZOH fiber versus the control group, showing the cell viability of the ZOH fiber reached 106.1%. Figure S13 shows the fluorescence images of live and dead cells after staining. Overall, the ZOH fiber exhibited excellent biocompatibility.

**Figure 5:**
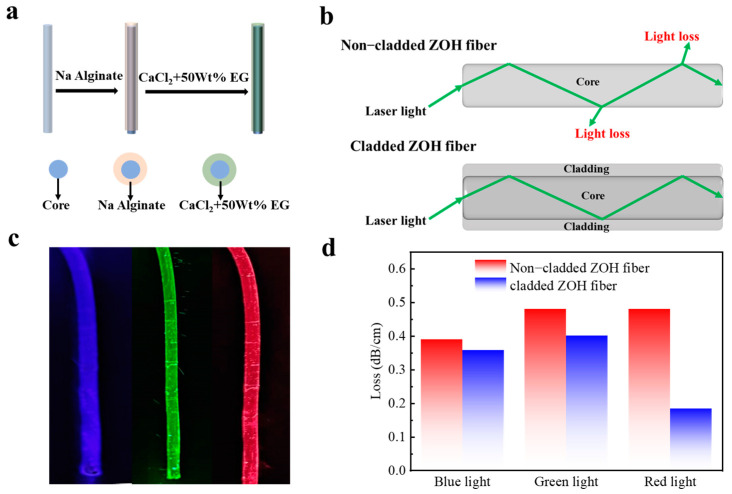
(**a**) Scheme of the fabrication of the core-coated ZOH optical fiber. (**b**) Schematic of the light transmission difference between the non-coated and the coated ZOH fiber. (**c**) Photos of lasers of different wavelengths passing through the coated ZOH fiber. (**d**) Optical transmission loss of lasers with different wavelengths passing through the non-coated and the coated ZOH fiber.

More importantly, ZOH fibers have excellent light-guiding performance and can be used as optical fibers and flexible nerve interfaces [40,41]. Importantly, high optical transparency enables low loss of optical waveguides, and the ZOH fibers have an optical transparency of 92.1%. However, light propagating into the fiber will be scattered, resulting in a higher light loss. Accordingly, we prepared a coated ZOH fiber by a two-step method (Figure 5a). Briefly, the prepared ZOH fibers were immersed in sodium alginate for one minute and then removed and immersed in a mixed solution of calcium chloride and 50 wt% EG for a period of time. Finally, coated ZOH fibers were obtained. Figure 5b shows the light transmission mechanism of the non-coated ZOH fiber and the coated ZOH fiber. It can be seen that after coating, the light is reflected to the coating and then reflected back into the fiber; therefore, the light transmission loss is greatly reduced.

As shown in Figure 5c, we used different wavelength lasers (blue: 450 nm, green: 532 nm, and red: 650 nm) to penetrate the coated ZOH fiber, using the uncoated ZOH fiber as a control (Figure S14). It can be seen that the light-guiding performance of the ZOH fiber after coating is better than that of the non-coated ZOH fiber. In order to clarify the loss of light transmission, we took an 8 cm long ZOH fiber and measured the relevant data with an optical power meter to calculate the light loss after different wavelengths of laser were shone on the fiber. As can be seen in Figure 5d, the largest difference in optical loss between the uncoated and coated ZOH fibers was observed when shining the red laser light onto the ZOH fiber. The light attenuation of the non-coated hydrogel was 0.48 dB/cm, while the light attenuation of the coated hydrogel was 0.17 dB/cm; therefore, the light transmission loss after coating was less. As a result of the excellent light-guiding performance of the ZOH fiber, it can be applied in the next generation of optical fiber sensors and flexible neural interfaces.

In addition, we have compared the ZOH fiber with recently reported gel fibers (Table 2) [42,43,44,45,46,47,48,49]. For example, the ZOH fibers have high elongation at break, high electrical conductivities and low light transmission loss compared to BC hydrogel fibers. Although the HEA/SA/PEGDA hydrogel fiber has a high transparency, it has a low elongation at break compared to the ZOH fiber. Overall, the ZOH fibers have attractive properties and have great potential in bioelectronics.

## 4. Conclusions

In summary, we prepared ZOH fibers by a combination of the mold method and the solvent replacement method. The synthesized ZOH fibers have high tensile properties and high ionic conductivity. In addition, a strain sensor based on the ZOH fiber exhibited high durability and long-term stability (the ZOH fiber could still maintain a good performance after 1000 anti-fatigue tests). Due to the low interfacial impedance of the ZOH fiber, it can be used as a bioelectrode to collect ECG and EMG signals. At the same time, due to its unique high transmittance (92.1%), the prepared coated ZOH fiber has low light propagation loss and is expected to be used in optical fibers and nerve interfaces.

## Figures and Tables

**Table 1:** Full names and abbreviations of materials and tests in the article.

Full Name	Abbreviation
Carboxylbetaine methacrylate	CBMA
Hydroxyethyl methacrylate	HEMA
[2-(methacryloyloxy) ethyl] dimethyl-(3-sulfopropyl) ammonium hydroxide	SBMA
Ethylene glycol	EG
Dimethyl sulfoxide	DMSO
Zwitterionic organohydrogel	ZOH
Zwitterionic hydrogel	ZH
Electrocardiogram	ECG
Electromyogram	EMG
Sodium alginate	SA
Lithium chloride	LiCl
Acrylamide	AAm
N, N’-methylenebisacrylamide	MBAA
2-hydroxy-4’-(2-hydroxythoxy)-2-methylpropiophenone	I2959
Differential scanning calorimetry	DSC

**Table 2:** Comparison of the ZOH fiber with recently reported gel fibers.

Materials	Breaking Elongation (%)	Ion Conductivity (S/m)	Transparency (%)	Optical Loss (dB/cm)	Reference
**ZOH fiber**	**905.8**	**0.54**	**92.1**	**0.17**	**This work**
PEGDA/AAm	63	-	94.1	0.18	42
BC	84	0.03	80~90	5.1	43
AAm/PEGDA/CA	-	-	-	0.28	44
AAm/SBMA/[EMIM][DCA]	1100	2	90	-	45
HEA/SA/PEGDA	~400		>90		46
AAm/SA	730	-	-	0.45	47
PAAS/PMA	1180 ± 100	1.8	-	-	48
PVA/HEC	400.3	-	-	-	49

## Data Availability

Not applicable.

## Supplementary Materials

The following supporting information can be downloaded at: https://www.mdpi.com/article/10.3390/bios13010115/s1, Figure S1. The FTIR spectra of the PAAm-SA hydrogel fiber, ZH fiber, and ZOH fiber. Figure S2. Young’s modulus of the ZOH fiber with different monomer ratios (SBMA to AM). Figure S3. Cyclic tensile stress–strain curves of the ZOH fiber with strains of 50%, 100%, 200%, 300%, and 400%. Figure S4. Compliance of the ZOH fiber on substrates. Figure S5. (a,b) Photographs demonstrating the stretchability of the ZOH fiber and the ZH fiber at −35 °C. Figure S6. The TG curves of the ZH fiber and the ZOH fiber. Figure S7. Ionic conductivity of the ZOH fiber with different LiCI concentrations. Figure S8. Relative resistance changes in the ZOH fiber-based sensor at around 100% strain at (a) room temperature (25 °C), (b) low temperature (−35 °C), and (c) high temperature (60 °C). Figure S9. Photos of bioelectrical signals of the tested ZOH fiber. Figure S10. EMG signal of a knee bend detected by ZOH fiber-based electrodes. Figure S11. The positions of ZOH fiber-based electrodes. Figure S12. Cell viability of the ZOH fiber and control group. Figure S12. Fluorescence images of dead cells (red) and living cells (green) after being cultured on different substrates after 24 h. Figure S14. Photos of lasers of different wavelengths passing through the non-coated ZOH fiber.
