# Supplementary material for: High-Performance Zwitterionic Organohydrogel Fiber in Bioelectronics for Monitoring Bioinformation
Source: Biosensors (Basel). 2023 Jan 9;13(1):115. doi: 10.3390/bios13010115 (PMC9855821; doi:10.3390/bios13010115)
Supplement: Supplementary file 1 [file biosensors-13-00115-s001.zip › biosensors-2092157-supplementary.pdf]

Supporting Information

High-Performance Zwitterionic Organohydrogel Fiber in Bioelectronics for Monitoring Bioinformation

Jun Xia, Jiabei Luo, Boya Chang, Chuanyue Sun, Kerui Li, Qinghong Zhang, Yaogang Li, Hongzhi Wang and Chengyi Hou

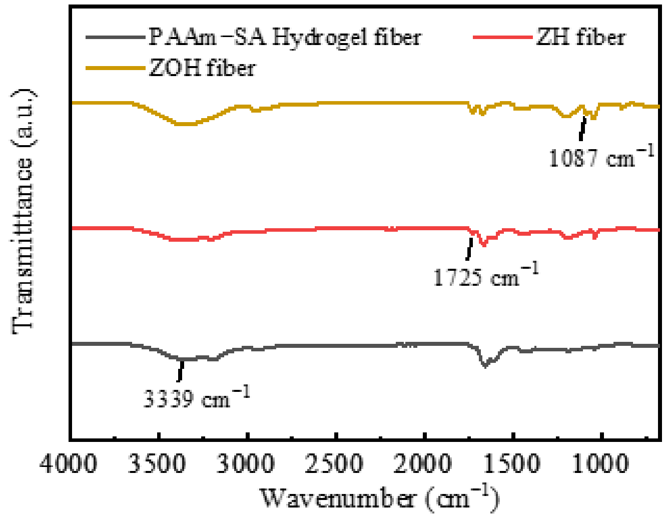

Figure S1. The FTIR spectrum of PAAm−SA Hydrogel fiber, ZH fiber and ZOH fiber.

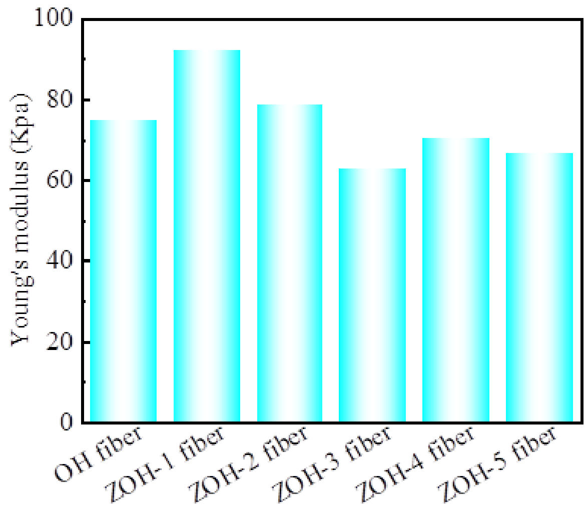

Figure S2. Young's modulus of the ZOH fiber with different monomer ratio (SBMA to AM).

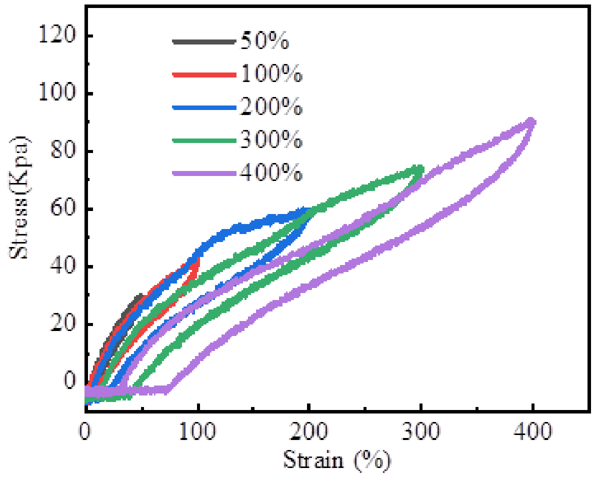

Figure S3. Cyclic tensile stress–strain curves of the ZOH fiber with strains of 50%, 100%, 200%, 300%,400%.

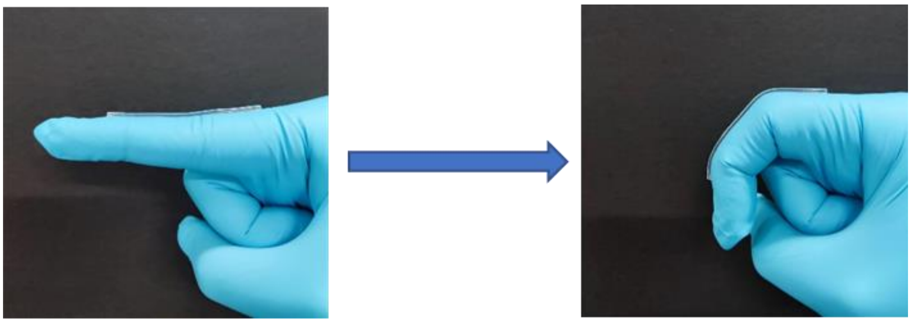

Figure S4. Compliance of the ZOH fiber on substrates.

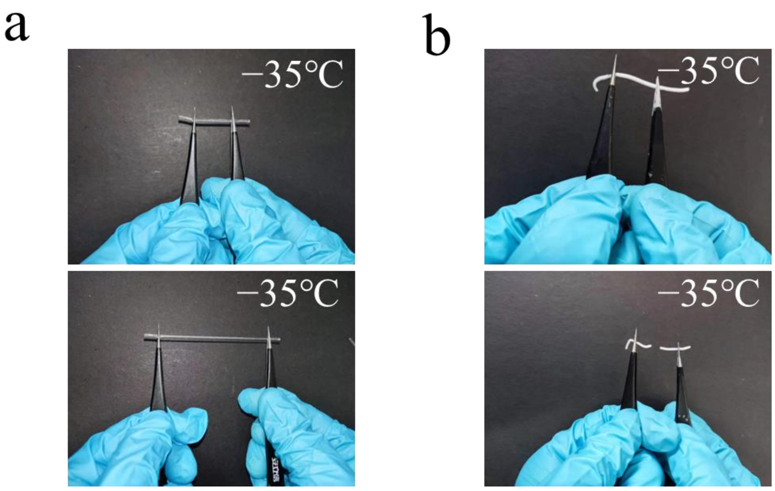

Figure S5. (a, b) Photographs demonstrate the stretchability of the ZOH fiber and the ZH fiber at −35°C.

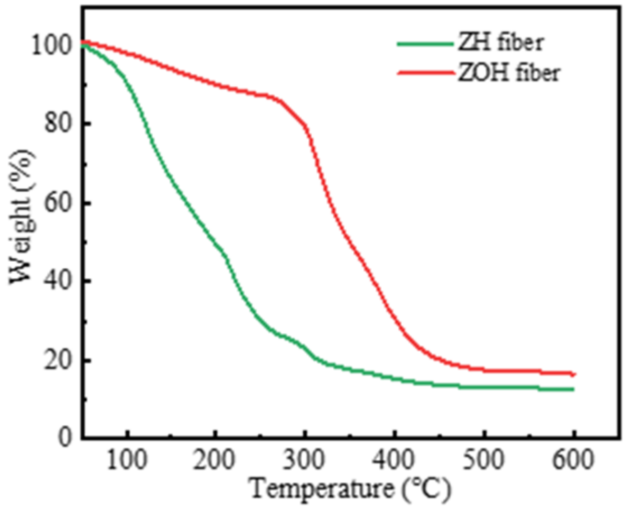

Figure S6. The TG curves of the ZH fiber and the ZOH fiber.

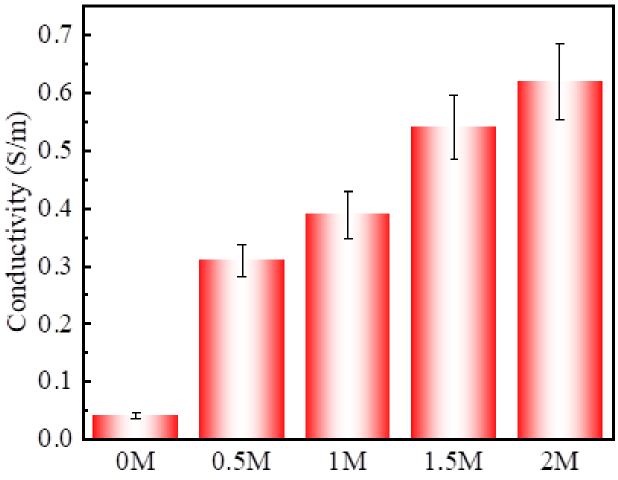

Figure S7. Ionic conductivity of the ZOH fiber with different LiCl addition.

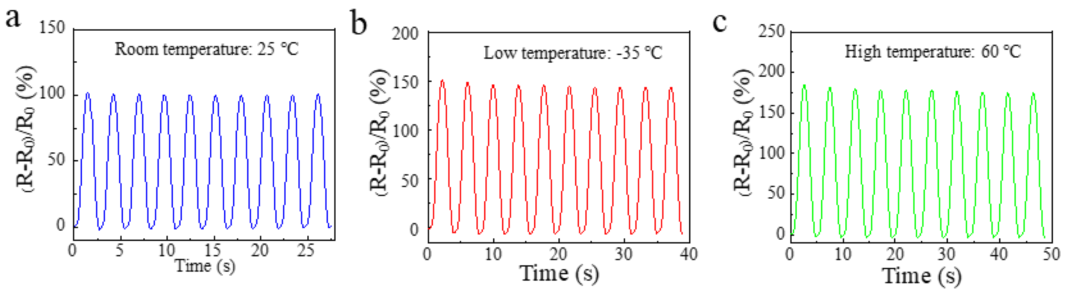

Figure S8. Relative resistance changes of the ZOH fiber-based sensor on around 100% strain at (a) room temperature (25°C), (b) low temperature (-35°C) (c) high temperature (60°C).

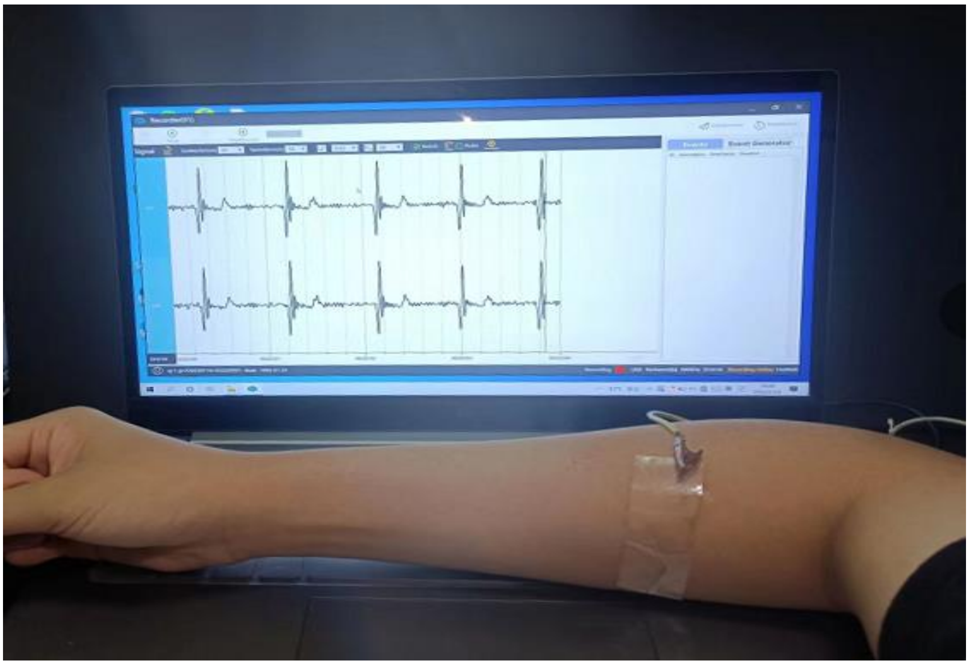

Figure S9. Photos of bioelectrical signals tested by the ZOH fiber.

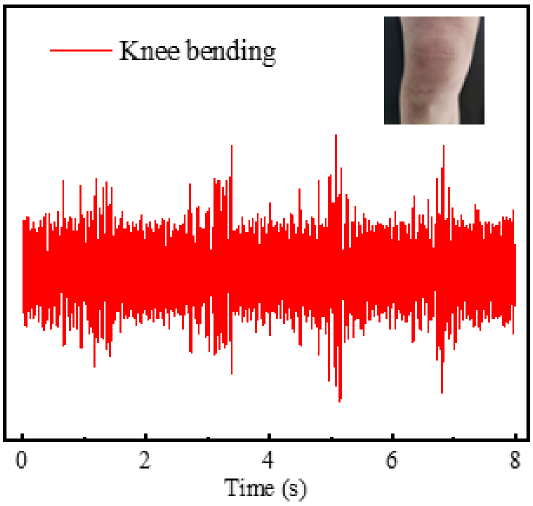

Figure S10. EMG signal of knee bending detected by ZOH fiber-based electrodes.

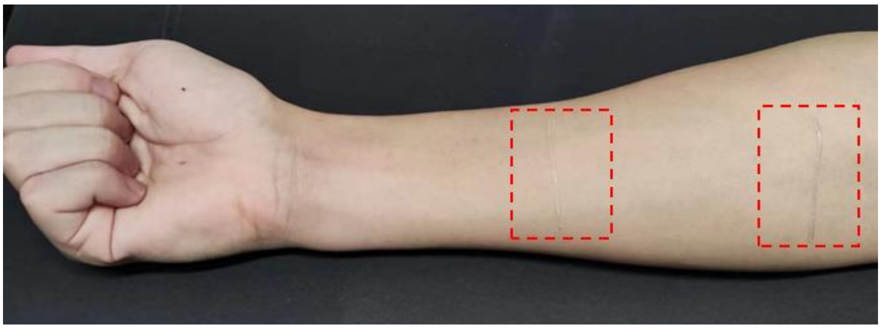

Figure S11. The positions of ZOH fiber-based electrodes.

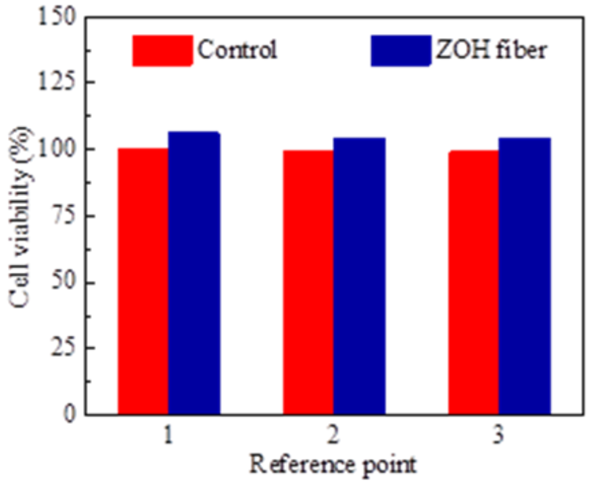

Figure S12. Cell viability of the ZOH fiber and control group.

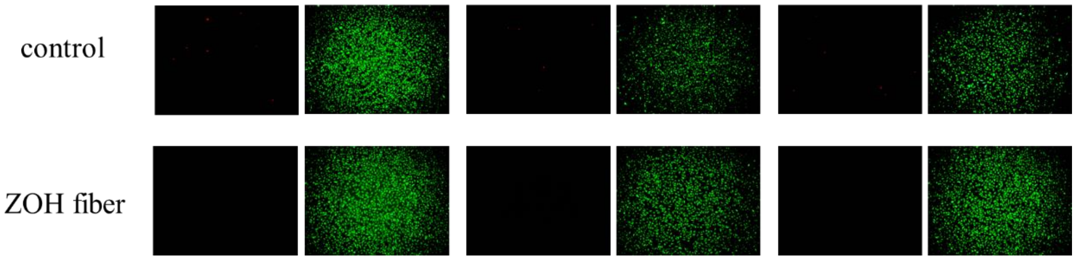

Figure S13. Fluorescence images of dead cells (red) and living cells (green) after cultured on different substrates after 24 hours.

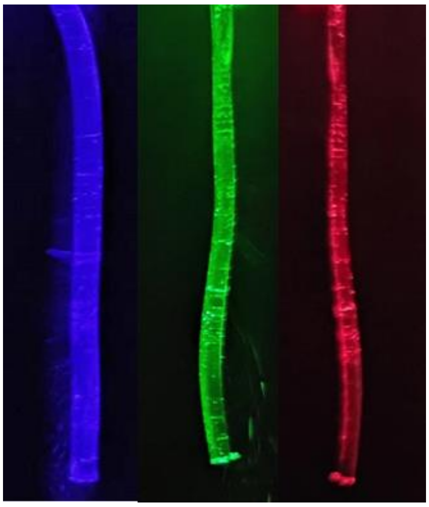

Figure S14. Photos of lasers of different wavelengths passing through the non-cladded ZOH fiber.
